# Gene Expression Profile of Isolated Dermal Vascular Endothelial Cells in Keloids

**DOI:** 10.3389/fcell.2020.00658

**Published:** 2020-07-29

**Authors:** Noriko M. Matsumoto, Masayo Aoki, Yuri Okubo, Kosuke Kuwahara, Shigeyoshi Eura, Teruyuki Dohi, Satoshi Akaishi, Rei Ogawa

**Affiliations:** ^1^Department of Plastic, Reconstructive and Aesthetic Surgery, Nippon Medical School, Tokyo, Japan; ^2^Department of Biochemistry and Molecular Biology, Nippon Medical School, Tokyo, Japan; ^3^Department of Plastic Surgery, Nippon Medical School Musashi Kosugi Hospital, Kawasaki, Japan

**Keywords:** keloid, wound healing, vascular endothelial cells, LAMC2, SERPINA3

## Abstract

Wound healing is a complex biological process, and imbalances of various substances in the wound environment may prolong healing and lead to excessive scarring. Keloid is abnormal proliferation of scar tissue beyond the original wound margins with excessive deposition of extracellular matrix (ECM) and chronic inflammation. Despite numerous previous research efforts, the pathogenesis of keloid remains unknown. Vascular endothelial cells (VECs) are a major type of inductive cell in inflammation and fibrosis. Despite several studies on vascular morphology in keloid formation, there has been no functional analysis of the role of VECs. In the present study, we isolated living VECs from keloid tissues and investigated gene expression patterns using microarray analysis. We obtained 5 keloid tissue samples and 6 normal skin samples from patients without keloid. Immediately after excision, tissue samples were gently minced and living cells were isolated. Magnetic-activated cell sorting of VECs was performed by negative selection of fibroblasts and CD45^+^ cells and by positive selection of CD31^+^cells. After RNA extraction, gene expression analysis was performed to compare VECs isolated from keloid tissue (KVECs) with VECs from normal skin (NVECs). After cell isolation, the percentage of CD31^+^ cells as measured by flow cytometry ranged from 81.8%–98.6%. Principal component analysis was used to identify distinct molecular phenotypes in KVECs versus NVECs and these were divided into two subgroups. In total, 15 genes were upregulated, and 3 genes were downregulated in KVECs compared with NVECs using the *t*-test (< 0.05). Quantitative RT-PCR and immunohistochemistry showed 16-fold and 11-fold overexpression of *SERPINA3* and *LAMC2*, respectively. SERPINA3 encodes the serine protease inhibitor, α1-antichymotripsin. Laminin γ2-Chain (LAMC2) is a subunit of laminin-5 that induces retraction of vascular endothelial cells and enhances vascular permeability. This is the first report of VEC isolation and gene expression analysis in keloid tissue. Our data suggest that *SERPINA3* and *LAMC2* upregulation in KVECs may contribute to the development of fibrosis and prolonged inflammation in keloid. Further functional investigation of these genes will help clarify the mechanisms of abnormal scar tissue proliferation.

## Introduction

Normal wound healing is a complex process composed of multiple sequential, albeit overlapping, phases (hemostasis, inflammatory, proliferative, and remodeling/maturation) that involve finely tuned interactions between many molecular, cellular, physiological, biochemical and mechanical factors (Berry et al., 1998; Wong et al., 2012; Aoki et al., 2019). When the remodeling/maturation phase is deranged, scars can start to grow and spread abnormally, thus resulting in the pathological scars known as keloids and hypertrophic scars (Diegelmann and Evans, 2004). Pathologically, these abnormal scars are characterized by excessive deposition of extracellular matrix (ECM) (Amadeu et al., 2004). To date, the mechanisms involved in the remodeling/maturation phase of normal and abnormal wound healing have been relatively poorly researched. This lack of knowledge has hampered the development of strategies that can prevent abnormal scar formation and/or effectively treat these scars once they have formed (Lee and Jang, 2018).

Multiple cell types play key roles in normal and abnormal wound healing: of particular interest are immune cells, fibroblasts, myofibroblasts, vascular endothelial cells (VECs), and mural cells (vascular smooth muscle cells and pericytes). In relation to immune cells, it is thought that abnormal prolongation of the inflammation into the scar remodeling phase is a crucial mechanism that underlies pathological scar formation and progression (Brissett and Sherris, 2001; Ogawa, 2017). Similarly, fibroblasts and myofibroblasts, which lay down ECM, are major players in the excessive fibrosis that characterizes pathological scarring. Finally, VECs and their extensive interactions with the surrounding mural cells appear to play key inductive roles in scar pathogenesis: there is now considerable evidence that their abnormal functions and interactions with immune and fibrotic cells may promote both the inflammation and ECM deposition in abnormal scarring. For example, in keloids, the metabolism of the ECM is profoundly abnormal, resulting in ‘keloidal collagen’, which are thick and complex collagen bundles that eventually become extremely interconnected (Ehrlich et al., 1994; Matsumoto et al., 2017). These pathological changes are accompanied by deranged angiogenesis, as shown by low capillary density, microvessel occlusion and luminal flattening, and a chaotic microvessel architecture characterized by relatively large capillaries with few connections with the smaller capillaries. Despite the relatively high blood flow in keloids (as shown by Doppler), it is likely that this faulty angiogenesis leads to local hypoxia that in turn promotes aberrant ECM metabolism (Tuan et al., 2008; Kurokawa et al., 2010). This hypothesis is supported by the fact that two effective treatments for keloids, namely, radiotherapy and laser therapy, act by suppressing angiogenesis and by destroying the poorly functioning blood vessels; the latter promotes the function of the stable blood vessels and thereby eliminates hypoxia (Park et al., 2012; Ogawa and Akaishi, 2016).

There are also other indirect lines of evidence that suggest that VEC dysfunction plays an important role in keloid pathogenesis (Ogawa and Akaishi, 2016). For example, patients with hypertension tend to have worse keloids than nonhypertensive patients (Arima et al., 2015). Moreover, patients with keloids have lower endothelial function than patients without keloids, as measured by reactive hyperemia peripheral arterial tonometry (Noishiki et al., 2017). It is also possible that the familial tendency of keloids and the fact that black people develop keloids much more readily than Caucasians relate to the presence of one or more single nucleotide polymorphisms that promote VEC dysfunction: one recent candidate is a variant of the N-acylsphingosine amidohydrolase-1 (ASAH1) gene, which is expressed by VECs and mural cells (Santos-Cortez et al., 2017).

Despite these observations, there are few functional analyses of the VECs from keloids. This reflects technical difficulties associated with isolating endothelial cells from tissues with excessive fibrosis. As a result, the roles of VECs in keloid formation and progression and their underlying molecular mechanisms remain poorly understood. We recently found a method for successfully isolating live endothelial cells from keloid tissues: the tissue is first gently digested and the resulting free cells are separated by magnetic sorting. The aim of this study was to analyze the gene expression profiles of keloid VECs to identify the factors that could contribute to (i) keloid formation and (ii) pathological interactions between angiogenesis, inflammation, and fibrosis.

## Materials and Methods

### Patients and Tissues

Tissue samples were obtained during surgical procedures that were performed between May 2016 and October 2016 at Nippon Medical School Hospital, Tokyo, Japan. Keloid tissues were obtained from the central region of surgically resected keloids. Normal skin samples were obtained from the ‘dog ear’ tissues (bulges of excess skin) that were left after reconstructive surgery and that had to undergo additional resection in patients without keloids or any systemic complications. The study was approved by the Institutional Review Board of Nippon Medical School, Tokyo, Japan and adhered to the principles of the Declaration of Helsinki and its revisions (27-02-560). Written informed consent was obtained from each study participant.

### Tissue Dissociation

The resected tissue specimens were placed in Dulbecco’s modified Eagle’s medium (DMEM; Fujifilm Wako, Osaka, Japan) supplemented with Antibiotic-Antimycotic (ABAM; Thermo Fisher Scientific, Waltham, MA, United States). The specimens were transported to the laboratory and cut into 1 × 2-cm with full thickness. The VECs were then isolated by washing the tissues three times with DMEM supplemented with ABAM, mincing the tissues into approximately 3-mm^3^ pieces with scissors, and digesting the mix with Skin Dissociation Kit (Miltenyi Biotech, Bergish Gladbach, Germany) according to the manufacturer’s instructions. Briefly, this process involved placing the tissue mix in a gentleMACS C tube together with DMEM containing 1% bovine serum albumin (BSA), Enzyme P, Enzyme D, and Enzyme A (Miltenyi Biotech). The mixture was then allowed to digest for approximately 1 hour in a GentleMACS Octo Dissociator with Heaters (Miltenyi Biotech). Subsequently, the cells were squeezed through 100-μm Cell Strainers (Corning, Corning, NY, United States) and harvested by centrifugation (300 *g*, 10 min, 4°C). Thereafter, PEB buffer composed of phosphate-buffered saline (PBS), pH 7.2, 0.5% BSA, and 2 mM EDTA was added to the cells and mixed well.

### Magnetic Sorting of VECs

**FIGURE 1:**
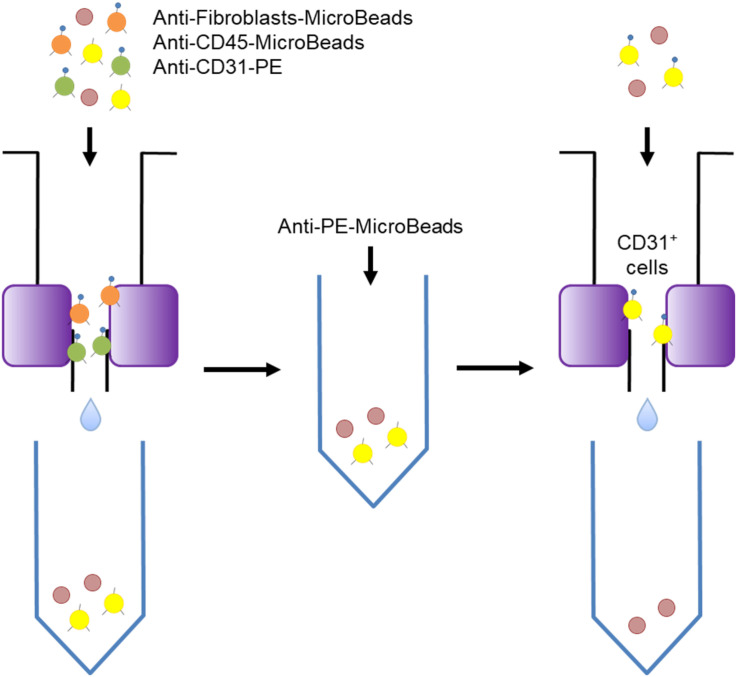
Schematic depiction of the magnetic sorting procedure used to isolate the skin vascular endothelial cells. Magnetic sorting was performed in two steps that separated the CD31-positive cells from the fibroblasts and CD45-positive cells.

The isolated cells were sorted by the Miltenyi Biotech magnetic sorting system as shown in Figure 1. The first step was to add Anti-CD45 MicroBeads and Anti-Fibroblast MicroBeads (the latter are conjugated to monoclonal mouse anti-human fibroblast antibodies and mainly target CD44) (Miltenyi Biotech) to the cells and to incubate the mixture at room temperature for 10 min. Anti-CD31-PE antibodies (Miltenyi Biotech) were then added, followed by incubation at 4°C in the dark. Subsequently, the cell suspension was subjected to magnetic sorting using LS Columns (Miltenyi Biotech) that had been washed three times with PEB. The cells that passed through the column were collected and centrifuged (300 *g*, 10 min, 4°C), after which the supernatant was removed. Anti-PE-MicroBeads (Miltenyi Biotech) and PEB were then added to the cells and mixed well. The resulting cell suspensions were loaded onto thrice-washed LS Columns and the cells that passed through were discarded. After removing the column from the magnet, the cells were washed out into a tube and then, to increase the purity of the VECs, passed through another LS column. Thereafter, the resulting suspension was immediately placed in RNAlater (Thermo Fisher Scientific). Some of the suspension was analyzed by flow cytometry using FACScan (BD Biosciences, Franklin Lakes, NJ, United States) to determine the purity of the VECs.

### RNA Extraction

Vascular endothelial cells RNA was isolated by using RNeasy Mini kit (Qiagen, Hilden, Germany) according to the manufacturer’s instructions. The integrity and overall quality of the total RNA were checked by using Agilent 2100 Bioanalyzer (Agilent Technologies, Santa Clara, CA, United States).

### Microarray Analysis

Vascular endothelial cells gene expression analysis was performed by using Agilent SurePrint G3 Human Gene Expression 8 × 60 K ver3.0 (Agilent Technologies) according to the manufacturer’s instructions (One-Color Microarray-Based Gene Expression Analysis Protocol Version Jan. 2012; Agilent Technologies). In brief, cyanine-3 (Cy3)-labeled cRNA was prepared from 75 ng RNA by using the One-Color Low Input Quick Amp labeling kit (Agilent Technologies. Dye incorporation and cRNA yield were checked with a NanoDrop spectrophotometer (Thermo Fisher Scientific). For hybridization, 600 ng of Cy3-labeled cRNA was fragmented at 60°C for 30 min. On completion of the fragmentation reaction, 25 μl of 2X Agilent hybridization buffer was added to the fragmentation mixture and hybridized for 17 h at 65°C in a rotating Agilent hybridization oven. After hybridization, the microarrays were washed with GE Wash Buffer. Slides were scanned after washing on the Agilent Technologies Microarray scanner. The fluorescence intensities on scanned images were extracted and preprocessed by Agilent Feature Extraction software (v10.7.3.1). The raw signals were normalized using the percentile shift normalization method; the value was set at 75th percentile and log-transformed. Universal Human Reference RNA (Agilent Technologies) was used as the control.

### Quantitative RT-PCR (qRT-PCR)

cDNA was synthesized using the High-Capacity cDNA Reverse Transcription Kit (Thermo Fisher Scientific). qRT-PCR assays were performed using an ABI Prism 7500 System (Applied Biosystems, Foster City, CA, United States) with Applied Biosystems TaqMan Gene Expression Assays (SERPINA3, Hs00153674_m1; LAMC2, Hs01043717_m1; GAPDH, Hs03929097_g1) and TaqMan Gene Expression Master Mix (Thermo Fisher Scientific). For each primer set, the optimal dilution was determined and melting curves were used to determine the amplification specificity. GAPDH served as the internal control after it was found to validate several expression assays. Relative expression was calculated by using the 2^–ΔΔCt^ method with correction for different amplification efficiencies.

### Immunohistochemistry

Formalin-fixed paraffin-embedded slices of the keloid and normal tissues were baked for 30 min at 58°C, deparaffinized in xylene, rehydrated through graded alcohol, and antigen-retrieved at 98°C in a water bath for 30 min in sodium citrate buffer (10 mM sodium citrate, pH 6.0). The slides were then immersed in 0.3% hydrogen peroxide diluted in methanol for 20 min to block endogenous peroxidase activity. Subsequently, they were preincubated with 5% bovine serum albumin (BSA) at 15–20°C for 15 min to reduce non-specific reactions. Thereafter, the slides were incubated with anti-SERPINA3 (1:50, Abcam, Cambridge, MA, United States) or anti-LAMC2 (1:200, GeneTex, Irvine, CA, United States) antibody. The slides were sequentially incubated with a secondary antibody, Histofine Simple Stain MAX-PO (MULTI) (Nichirei Biosciences, Tokyo, Japan) at 15–20°C for 30 min, followed by staining with DAB (Dako, Glostrup, Denmark). Finally, the slides were counterstained with Mayer’s hematoxylin, dehydrated in graded concentrations of ethanol, and mounted.

### Lipopolysaccharide (LPS) Stimulation of a Dermal VEC Line

The HMEC-1 cell line is derived from human dermal microvascular endothelium. It was purchased from ATCC (Manassas, VA, United States) and cultured in MCDB131 medium supplemented with 10 ng/mL epidermal growth factor, 1 μg/mL hydrocortisone, 10 mM glutamine, and 10% fetal bovine serum (FBS). LPS from E. coli O111 was purchased from Fujifilm Wako (Osaka, Japan). The HMEC-1 cells were plated in a 24-well plate at densities of 4 × 10^4^ cells/well and grown until sub-confluency. The cells were then cultured with or without 1 μg/ml LPS for 15 h (Dayang et al., 2019), after which the cells were harvested for RNA extraction and qRT-PCR.

### Effect of Laminin-5 on Dermal Fibroblast Proliferation and Apoptosis

Laminin-5 consists of α, β, and γ subunits; the γ subunit is encoded by LAMC2 (Hashimoto et al., 2006). Normal human dermal fibroblasts (NHDFs) were purchased from TaKaRa (Shiga, Japan) and cultured in Dulbecco’s Modified Eagle’s Medium (DMEM) supplemented with 10% FBS. They were plated in 96-well plates at density of 1 × 10^4^ cells/well and grown for 24 h. The cells were then treated with or without 0.5 μg/ml of human recombinant laminin-5 (also known as laminin-332) (Oriental Yeast, Tokyo, Japan) in DMEM containing 2% FBS for 15 h (Baba et al., 2008). Cell proliferation and apoptosis were assessed by using ApoLive-Glo Multiplex Assay (Promega, Madison, WI, United States) according to the manufacturer’s instructions. Thus, the cells were incubated with Viability Reagent for 30 min at 37°C and fluorescence was measured at the wavelength set of 400Ex/505Em. The cells were then incubated with Caspase-Glo 3/7 Reagent for 30 min at room temperature and luminescence was measured.

### Statistical Analysis

Comparisons between the two groups in terms of continuous variables were performed with Student’s *t*-test (if the data were normally distributed) or with the non-parametric Mann-Whitney-Wilcoxon (if the data were not normally distributed). Statistical significance was set at *p* < 0.05, unless otherwise specified. Statistical analyses were performed with EZR (Saitama Medical Center, Jichi Medical University, Saitama, Japan), which is a graphical user interface for R 2.13.0 (R Foundation for Statistical Computing, Vienna, Austria). More precisely, EZR is a modified version of R commander (version 1.6-3) that was designed to add statistical functions that are frequently used in biostatistics.

## Results

### Efficiency and Accuracy of VECs Isolation and RNA Extraction

**FIGURE 2:**
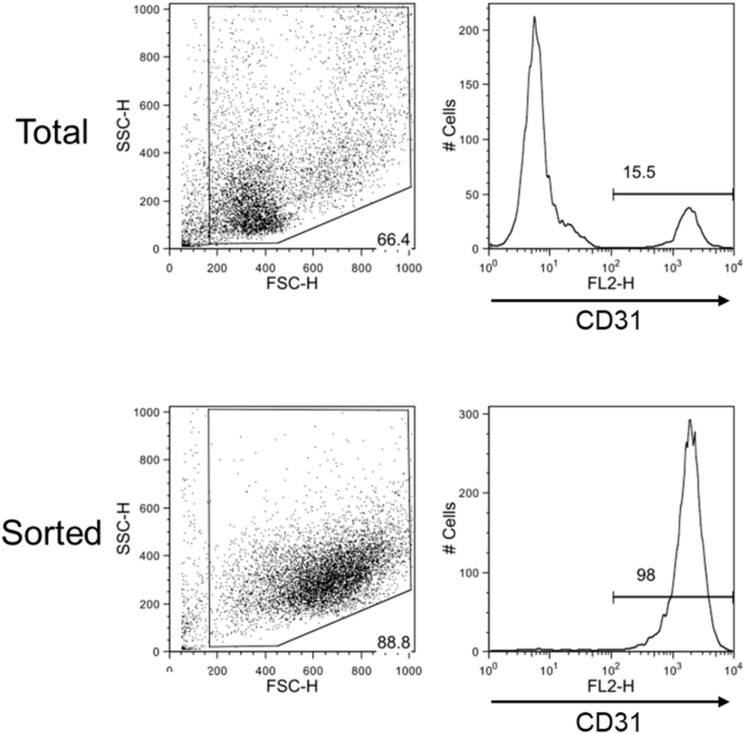
Flow cytometric evaluation of the efficiency with which skin vascular endothelial cells were isolated. Representative flow cytometry plots and histograms of total dispersed cells before sorting (upper) and after sorting (lower) are shown. Very pure CD31-positive cell samples were obtained by magnetic sorting.

The magnetic sorting system shown in Figure 1 successfully and efficiently isolated live VECs from keloid and normal skin samples. Total RNAs were then obtained from the VECS of five keloid samples (KVECs) and six normal skin samples (NVECs). The clinical characteristics of the patients, the purity of the isolated VECs (as measured by flow cytometry), and the integrity of the extracted RNAs are presented in Table 1. VEC purity ranged from 81.8% to 98.6%. Representative flow cytometric plots and histograms are shown in Figure 2. RNA integrity (as expressed by RNA Integrity Number) ranged between 6.4 and 9.4.

**TABLE 1:** Characteristics of the patients, vascular endothelial cell (VEC) purity, and RNA integrity.

Sample No.	Range of age	Area	VECs (% of cells in the sample)	RIN
**Keloids**				
K-1	26–30	Shoulder	97.5	8.6
K-2	60–65	Anterior chest	93.4	8.8
K-3	60–65	Lower abdomen	98.6	9.4
K-4	20–25	Upper limb	98.3	9.3
K-5	56–60	Lower abdomen	98.5	8.3
**Normal skin samples**				
N-6	76–80	Eyelid	85.9	7.5
N-7	70–75	Thigh	96.8	6.4
N-8	60–65	Dorsal	95.5	9.2
N-9	76–80	Eyelid	97.4	9.1
N-10	70–75	Eyelid	81.8	6.8
N-11	60–65	Lower abdomen	93.9	7.0

*RIN, RNA integrity number.*

### Microarray Analysis Followed by Hierarchical Clustering and Principal Component Analysis

**FIGURE 3:**
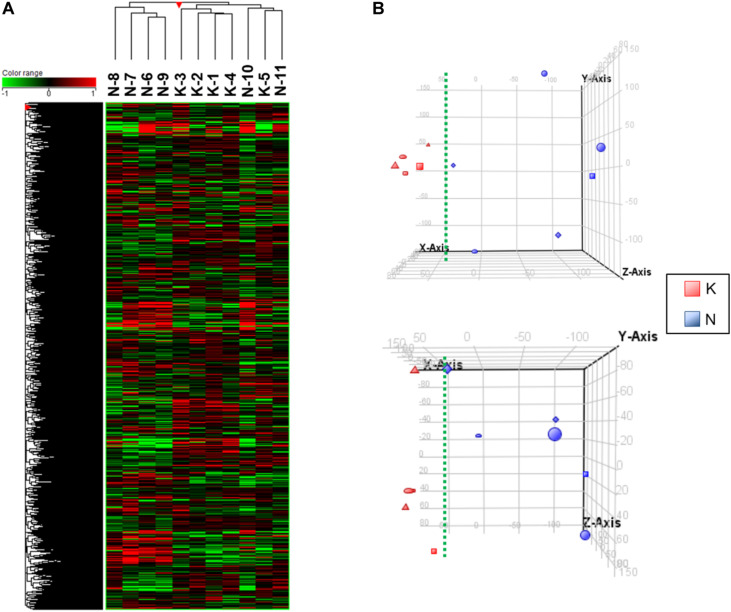
Hierarchical clustering and principal component analysis (PCA) of the vascular endothelial cell (VEC) samples. **(A)** The expression patterns of all samples are shown in the heatmap. The sample numbers of the normal VECs (NVECs) and keloid VECs (KVECs) are indicated at the top. On hierarchical clustering, the KVECs and NVECs generally fell into two separate clusters. **(B)** PCA plots that visualize the distribution of KVECs and NVECs in terms of gene expression pattern. The KVECs were distributed close to each other and away from the NVECs. As a result, the KVECs could be divided from the NVECs by the indicated green broken line. The upper and lower plots indicate the distributions from two different 3-dimensional viewpoints.

The VEC RNAs were subjected to microarray analysis followed by hierarchical clustering and principal component analysis (PCA): this allowed us to visualize significant gene expression differences between the KVECs and NVECs. Hierarchical clustering showed that the KVECs and NVECs largely fell into two separate clusters: the exception was one KVEC sample (K5), which formed a subcluster that branched out from the main KVEC cluster with two NVEC samples (N10 and N11). Thus, the mRNA expression profiles of the KVECs were largely different from those of the NVECs (Figure 3A). PCA then showed that the KVECs were located a marked distance away from the NVECs (Figure 3B, upper plot); this trend was confirmed by a different 3-dimensional representation of the data (Figure 3B, lower plot). Thus, while there was substantial sample variability, the KVECs clearly had different molecular phenotypes compared to the NVECs.

### Extraction of Genes and Network Analysis

**FIGURE 4:**
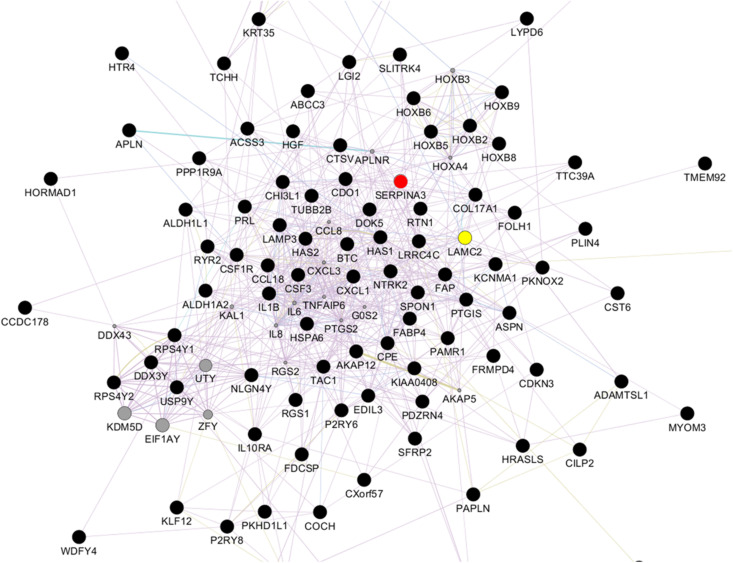
Network analysis with the 74 and 80 genes that were, respectively, up- and down-regulated by more than 5-fold in keloid vascular endothelial cells. SERPINA3 (red) and LAMC2 (yellow) are located near the center of the network.

Relative to NVECs, 15 and three genes in KVECs were respectively up- and down-regulated (as determined by Welch’s *t*-test; all *p* < 0.05) (Table 2). Table 3 lists the 10 genes that exhibited the greatest up- or downregulation in KVECs relative to NVECs. Of these, we focused on two factors, SERPINA3 and LAMC2, which are known to be involved in ECM metabolism and inflammation, respectively (Aumailley, 2013; Li et al., 2018). SERPINA3 and LAMC2 exhibited 16- and 11-fold upregulation in the microarray analysis, respectively (Table 3). To visualize whether these genes play an important role in KVEC gene regulation, we performed network analysis with the 74 and 80 genes that were, respectively, up- and down-regulated in KVECs by more than 5-fold. Both SERPINA3 and LAMC2 were located near the center of the network, which suggests that they may play important roles in KVEC phenotype (Figure 4).

**TABLE 2:** Genes that were expressed at significantly (*p* < 0.05) higher or lower levels in KVECs compared to in NVECs (as determined by using Welch’s *t-*test).

Probe Name	Gene Abbreviation	Gene Name	Corrected p-value
**Upregulated**			
A_23_P103256	CFHR3	complement factor H-related 3	0.010
A_33_P3304170	PIK3CG	phosphatidylinositol-4,5-bisphosphate 3-kinase, catalytic subunit gamma	0.010
A_23_P41145	FAM3D	family with sequence similarity 3, member D	0.018
A_33_P3367692	CFH	complement factor H	0.018
A_19_P00808586	LINC01085	long intergenic non-protein coding RNA 1085	0.018
A_33_P3286349	DNAAF3	dynein, axonemal, assembly factor 3	0.018
A_33_P3318288	CFH	complement factor H	0.018
A_23_P114740	CFH	complement factor H	0.025
A_32_P35969	CHRNA7	cholinergic receptor, nicotinic, alpha 7 (neuronal)	0.026
A_23_P49559	GPR142	G protein-coupled receptor 142	0.026
A_23_P79251	EHD3	EH-domain containing 3	0.026
A_33_P3333158	CPXM2	carboxypeptidase X (M14 family), member 2	0.026
A_23_P124927	RGS14	regulator of G-protein signaling 14	0.035
A_23_P57110	SLC52A3	solute carrier family 52 (riboflavin transporter), member 3	0.042
A_33_P3245631	TTC39A	tetratricopeptide repeat domain 39A	0.050
**Downregulated**			
A_33_P3245290	AQP7P1	aquaporin 7 pseudogene 1	0.020
A_21_P0005172	LINC00472	long intergenic non-protein coding RNA 472	0.026
A_22_P00017310	MIR99AHG	mir-99a-let-7c cluster host gene (non-protein coding)	0.044

*KVEC, keloid vascular endothelial cell; NVEC, normal vascular endothelial cell.*

**TABLE 3:** Genes that were highly up- or down-regulated in KVECs compared to in NVECs.

Probe Name	Gene Abbreviation	Gene Name	FC
**Upregulated**			
A_33_P3211198	NCMAP	noncompact myelin associated protein	30.51
A_23_P137797	RYR2	ryanodine receptor 2 (cardiac)	18.51
A_23_P2920	SERPINA3	serpin peptidase inhibitor, clade A (alpha-1 antiproteinase, antitrypsin), member 3	16.08
A_23_P160214	TTC39A	tetratricopeptide repeat domain 39A	12.57
A_23_P160968	LAMC2	laminin, gamma 2	11.01
A_19_P00323692	XIST	X inactive specific transcript (non-protein coding)	10.86
A_33_P3399788	SERPINA3	serpin peptidase inhibitor, clade A (alpha-1 antiproteinase, antitrypsin), member 3	10.81
A_23_P69154	FAM198A	family with sequence similarity 198, member A	10.75
A_23_P94902	KCTD8	potassium channel tetramerization domain containing 8	10.54
A_23_P29773	LAMP3	lysosomal-associated membrane protein 3	10.44
**Downregulated**			
A_23_P324384	RPS4Y2	ribosomal protein S4, Y-linked 2	−40.26
A_33_P3224331	DDX3Y	DEAD (Asp-Glu-Ala-Asp) box helicase 3, Y-linked	−23.37
A_23_P259314	RPS4Y1	ribosomal protein S4, Y-linked 1	−21.81
A_23_P15786	KRT25	keratin 25, type I	−19.70
A_32_P98072	TCHH	trichohyalin	−15.51
A_23_P99044	KRT71	keratin 71, type II	−15.21
A_23_P97141	RGS1	regulator of G-protein signaling 1	−13.98
A_23_P78201	KRT35	keratin 35, type I	−13.10
A_24_P234768	HTR4	5-hydroxytryptamine (serotonin) receptor 4, G protein-coupled	−11.24
A_23_P362694	FDCSP	follicular dendritic cell secreted protein	−10.19

*KVEC, keloid vascular endothelial cell; NVEC, normal vascular endothelial cell.*

### Validation of Microarray Data Regarding SERPINA3 and LAMC2 mRNA Expression

**FIGURE 5:**
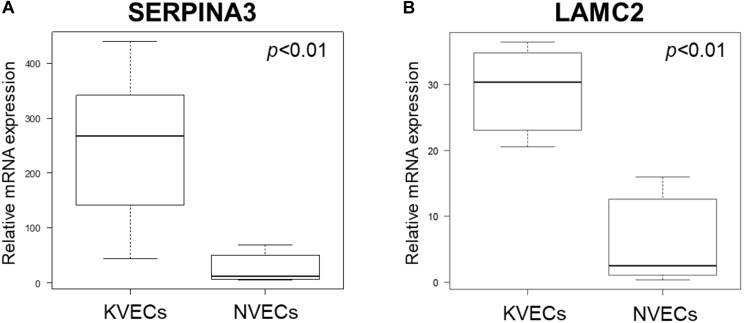
Validation of the microarray data by quantitative RT-PCR. The relative mRNA expression levels of SERPINA3 **(A)** and LAMC2 **(B)** in keloid vascular endothelial cells (KVECs) (*n* = 7) and normal vascular endothelial cells (NVECs) (*n* = 6) are shown. All values in this figure represent the mean ± SD.

We performed qRT-PCR to verify the microarray data. Given the relatively large variation between samples, qRT-PCR was performed using RNA extracted from six NVECs and seven KVECs: thus, two additional KVEC samples from different patients were added to the five KVEC samples that were used to generate the microarray analysis data. The two additional KVEC samples were from patients aged 36–40 years whose keloids were on the anterior chests (VEC purities were 98.8% and 99.1%). The qRT-PCR analysis confirmed that the KVECs had significantly higher mRNA expression levels of both SERPINA3 and LAMC2 compared to NVECs (Figures 5A,B).

### Histological Validation of SERPINA3 and LAMC2 Protein Expression in Blood Vessels of Skin Samples

**FIGURE 6:**
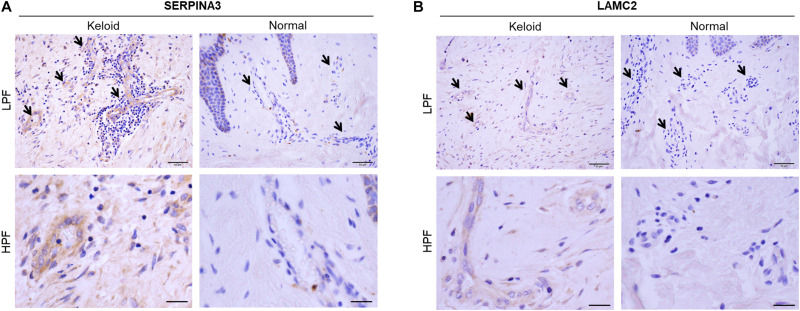
Immunohistochemical validation of **(A)** SERPINA3 and **(B)** LAMC2 protein expression in the blood vessels of keloid and normal skin tissues. Representative histological images are shown. Arrows indicate the blood vessels in the dermis. LPF, low-power field, HPF, high-power field. Scale bars: LPF, 50 μm, HPF, 20 μm.

Three keloids and three normal skin specimens were obtained by surgery from a separate cohort of patients. The keloid samples were obtained from patients whose keloids were on the lower abdomen (age 56–60 years) and anterior chests (age 61–65 and 76–80 years). The three non-keloid samples were from patients aged 11–15, 21–25, and 71–75 years. Immunohistochemical analysis of the six samples showed that all of the keloids had greater protein expression of both SERPINA3 and LAMC2 in the blood vessels than the normal skin samples. Representative images are shown in Figures 6A,B. These results are consistent with the microarray data.

### Inflammation-Induced Dysfunction Increases SERPINA3 and LAMC2 Expression in VECs but LAMC2 Does Not Affect Fibroblast Proliferation

**FIGURE 7:**
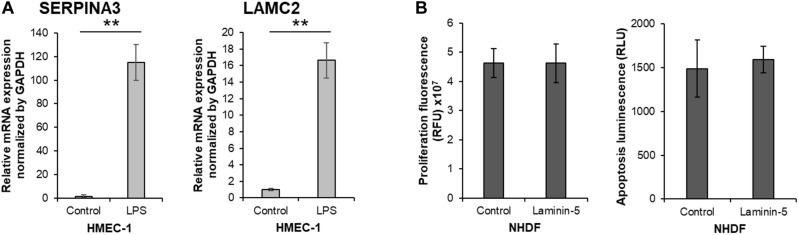
*In vitro* study on the biological functions of SERPINA3 and LAMC2 in vascular endothelial cells and fibroblasts. **(A)** A human dermal vascular endothelial cell line (HMEC-1) was treated with or without 1 μg/ml lipopolysaccharide (LPS) for 15 h (*n* = 6). The relative mRNA expression levels of SERPINA3 and LAMC2 in the cells was determined. **(B)** Normal human dermal fibroblasts (NHDF) were treated with or without 0.5 μg/ml laminin-5 for 15 h (*n* = 10). The relative cell proliferation and apoptosis was measured. All values in this figure represent the mean ± SD. ** *p* < 0.01.

To assess whether SERPINA3 and LAMC2 could participate in keloid pathogenesis, we asked whether activation with LPS changes the SERPINA3 and LAMC2 expression exposed to it, blood vessels exhibit hyperpermeability, and VECs adopt proinflammatory leukocyte-recruiting phenotypes, including the production of proinflammatory cytokines, chemokines, and adhesion molecules (Wang L. et al., 2017; Dayang et al., 2019). Indeed, LPS treatment of the VEC line with LPS caused it to upregulate SERPINA3 and LAMC2 expression (Figure 7A). This is consistent with the upregulated expression of these genes in KVECs (Figures 5, 6).

Since secreted LAMC2 promotes the proliferation and survival of cancer cells (Garg et al., 2014; Sato et al., 2014), and excessive fibroblast proliferation and insufficient apoptosis play an important role in keloid pathogenesis (Funayama et al., 2003), we speculated that LAMC2-overexpressing VECs could secrete LAMC2 and thereby promote local fibroblast proliferation and survival. However, we observed that the LAMC2-treated fibroblasts did not exhibit increased proliferation or resistance to apoptosis (Figure 7B).

## Discussion

Keloids and hypertrophic scars are caused by genetic and environmental factors; the environmental factors can be divided further into local and systemic factors. In terms of genetic factors, keloids associate with single nucleotide polymorphisms; in addition, people with dark skin are 15-fold more likely to develop keloids than people with lighter skin (Miller and Nanchahal, 2005; Nakashima et al., 2010). The local factors that promote pathological scar development are, like the genetic factors, well-studied and include the elastic tension on the skin and mechanical stress (Aoki et al., 2020). Much less well-understood are the systemic factors that associate with keloidogenesis; these include adolescence, pregnancy (Park and Chang, 2012; Krakowski et al., 2016), and endothelial dysfunction. With regard to the latter factor, mounting evidence now suggests that disturbed endothelial function may in fact play a key role in initiating and augmenting the pathogenic activities of other important cellular players in pathological scarring. For example, our recent studies show that poor endothelial function and hypertension associate with keloid development and aggravation (Arima et al., 2015; Noishiki et al., 2017). These clinical observations led us to analyze the gene expression in VECs from keloid patients, with the aim of identifying local cell interactions or microenvironments that could be exploited therapeutically.

The proper maintenance of vascular integrity requires that VECs interact with and adhere to the ECM. Along with endothelial cell proliferation and migration, these interactions between VECs and the ECM also play an important role in the angiogenesis that participates in crucial processes such as osteogenesis, tumor growth, and wound healing (Langen et al., 2017; Lugano et al., 2018; Bi et al., 2019). It is known that during the aberrant wound healing that leads to pathological scarring, VEC proliferation and new blood vessel formation may be shaped by immune and other factors such as endothelin-1, vascular endothelial growth factor, platelet-derived growth factor, and transforming growth factor-β. Notably, VECs may also secrete the latter factor, thereby activating nearby fibroblasts and initiating fibrotic reactions (Kiya et al., 2017; Wang X.Q. et al., 2017). This pathogenic role of VECs is supported by our finding that the abundant hyalinized keloidal collagen that characterizes keloids appear to arise from the perivascular area (Matsumoto et al., 2017). Based on these reports, we postulated that when inflammation occurs, VECs may play important roles in regulating the behavior of other cell types and tissue homeostasis. In the present study, we identified the genes that are up- or down-regulated in KVECs and then focused on SERPINA3 and LAMC2, both of which are sharply up-regulated in KVECs and thus may contribute to keloid pathogenesis.

The serpin family consists of a functionally diverse set of proteins that are named after their function as serine proteinase inhibitors (Potempa et al., 1994). Serpins play important roles in inflammation, immunity, coagulation, dementia, and cancer and can be potential biomarkers and therapeutic targets for disease (Heit et al., 2013). SERPINA3 is also known as α1-antichymotrypsin: it is an acute phase reactive protein and a serpin family member. Although its biological role in wound healing remains unknown, SERPINA3 appears to reduce leukocyte protease-mediated tissue damage (Gooptu and Lomas, 2009). Serpins also inhibit neutrophil-derived cathepsin G, which is a potent regulator of inflammatory processes and is directly involved in the degradation of ECM molecules that are essential for skin repair (Capodici et al., 1989), including collagen (Cipriani et al., 2018; Ito and Nagata, 2019), elastin (Ma et al., 2013), and fibronectin (Chang et al., 2012; Li et al., 2017). Furthermore, SERPINA3 is a potent inhibitor of matrix metalloproteinase type 9 activation in human wound healing (Han et al., 2008; Reiss et al., 2009). Our data showed that SERPINA3 is upregulated by 16-fold in KVECs compared to NVECs; this upregulation in VECs was observed at both the mRNA and protein level. Notably, we also found that when normal VECs become inflamed and dysfunctional due to exposure to LPS, their expression of SERPINA3 rises significantly. Thus, given its roles in inflammation, SERPINA3 may also act in keloid pathogenesis by altering local immune responses.

Laminins are large heterotrimers that constitute the basement membrane in ECM. They are composed of α, β, and γ subunits (Yurchenco et al., 2018). VECs attach to several ECM components, including fibronectin, collagen, and laminin (Kubota et al., 1988). Laminins not only directly affect leukocyte migration, they also indirectly influence endothelial barrier function by modulating endothelial barrier properties (Song et al., 2017). LAMC2 is the short arm of Laminin-5. It shapes cell motility and growth, thereby affecting morphogenic events (Navdaev et al., 2008; Garg et al., 2014). The targeted degradation of laminin-5 and LAMC2 by matrix metalloproteinases promote cell migration (Giannelli et al., 1997; Cheng et al., 2009). Significantly, LAMC2 induces the shrinkage of VECs, thereby enhancing vascular permeability *in vitro* and *in viv*o (Sato et al., 2014). Our data showed that KVECs have 11-fold upregulated expression of LAMC2. Moreover, as with SERPINA3, LPS treatment of normal VECs increased their LAMC2 expression. These findings together with the known activities of LAMC2 suggest that LAMC2 overexpression in KVECs may encourage endothelial hyperpermeability, which in turn may prolong the inflammatory stage, thereby promoting keloid formation and progression. Since LAMC2 promotes the proliferation and survival of cancer cells (Garg et al., 2014; Sato et al., 2014), and keloids are characterized by excessive fibroblast proliferation and resistance to apoptosis (Funayama et al., 2003), we also asked whether LAMC2 secretion by VECs could promote keloid fibroblast proliferation and downregulate apoptosis. However, culturing normal fibroblasts with LAMC2 had no effect on these properties. Further studies on the potential underlying mechanisms are warranted.

Because keloids are elastic fibrotic lesions that are formed by excessive deposition of ECM components, it is hard to digest keloids and isolate cells with small populations such as VECs. While VECs have been isolated from soft organs or VECs-rich tumors (Mao et al., 2016; Liu et al., 2017; Crouch and Doetsch, 2018), only one study has reported the isolation of VECs from (hypertrophic) scars (Wang et al., 2008). However, recent technological advances, namely, the application of gentle digestion followed by magnetic sorting, allowed us to isolate VECs from keloid tissues. The gentle digestion contributes to the high cell survival rates and the two magnetic cell sorting-based isolation steps that, respectively, removed the many CD45-positive cells and fibroblasts and positively selected the CD31-positive VECs led to very pure VECs populations. In the future, similar recent advances in this technology will be used to isolate and analyze targeted single cells.

This study has some limitations. First, not all VECs in keloids express CD31. Second, the up- and down-regulated genes do not simply reflect the activities of KVECs: rather, they are likely to reflect the sum of not only KVEC behavior but also systemic and local inflammatory factors. More functional experiments in which these genes are knocked out or down are required.

## Conclusion

This is the first study to analyze the expression profiles of VECs from keloids. It showed that two genes involved in immune cell, ECM, and endothelial regulation, namely, SERPINA3 and LAMC2, are highly upregulated in KVECs compared to in NVECs. These findings may not only help to clarify how keloids develop but they may also be useful targets for anti-keloid therapies that are effective at different stages after onset. For example, inhibiting LAMC2 early after onset may prevent the vascular permeability and prolongation of inflammation that drives keloid growth. Similarly, inhibiting SERPINA3 in well-established keloids may promote immune responses and the degradation of the ECM, thereby reducing the keloid mass and/or preventing its further outgrowth. More detailed functional analyses of the VECs in keloids are warranted.

## Data Availability Statement

The datasets generated for this study can be found in the microarray datasets submitted to Gene Expression Omnibus (GEO)-NCBI and are accessible through GEO Series accession number GSE121618.

## Ethics Statement

The studies involving human participants were reviewed and approved by The Institutional Review Board of Nippon Medical School, Tokyo, Japan. Written informed consent to participate in this study was provided by the participants’ legal guardian/next of kin.

## Author Contributions

NM collected, assembled, analyzed, interpreted the data, and wrote the draft of manuscript. MA designed the study, analyzed, interpreted the data, and wrote the manuscript. YO, KK, SE, and TD analyzed and interpreted the data. SA and RO supervised the study and performed critical review. All authors contributed to the article and approved the submitted version.

## Conflict of Interest

The authors declare that the research was conducted in the absence of any commercial or financial relationships that could be construed as a potential conflict of interest.
